# The impact of exercise interventions on cognitive frailty: a scoping review of outcomes and biological mechanisms

**DOI:** 10.3389/fpubh.2025.1738522

**Published:** 2026-01-12

**Authors:** Xiaofei He, Yuxiao Jiao, Lulu Ma, Bo Zhang, Lanyu Zhu

**Affiliations:** School of Nursing, Changchun University of Chinese Medicine, Changchun, China

**Keywords:** biological mechanisms, cognitive frailty, cognitive function, exercise, physical function

## Abstract

**Background:**

As the global trend of aging intensifies, the high prevalence of cognitive frailty is expected to impose a substantial burden on healthcare systems. While traditional exercise interventions have demonstrated effectiveness, contemporary models are evolving from conventional in-person formats toward digitally-supported, personalized approaches. Nevertheless, a comprehensive synthesis of evidence comparing the efficacy of different exercise modalities and elucidating their underlying biological mechanisms in the context of cognitive frailty remains lacking.

**Objective:**

This scoping review aims to elucidate a multidimensional integrated framework linking “exercise formats-mechanisms-health outcomes” for exercise interventions improving cognitive frailty, thereby systematically revealing the action pathways and synergistic mechanisms of different exercise modalities.

**Methods:**

This review adhered to the PRISMA-ScR guidelines and systematically searched PubMed, Cochrane Library, Embase, Web of Science, and Scopus up to October 9, 2025. Literature screening, data extraction, and synthesis were performed to identify studies investigating the effects of exercise interventions on cognitive frailty.

**Results:**

Seventeen studies were included. The results indicate that exercise intervention models are diverse, with nearly half of the studies employing digital intervention models, demonstrating new potential for enhancing safety, adherence, and personalization. An integrated analysis of seven studies reporting mechanistic insights indicated that the benefits are supported by multiple synergistic biological pathways, including improvements in brain structure and functional plasticity, enhanced neural efficiency, reduced neuroinflammation and oxidative stress, modulation of neurotransmitters, and increased cerebral blood flow perfusion.

**Conclusion:**

This scoping review systematically examines the integrated framework of “exercise formats-mechanisms-health outcomes” to reveal the multidimensional synergistic network through which exercise interventions ameliorate cognitive frailty, while also highlighting the considerable potential of the digital transformation of exercise modalities. Future research should deepen the integration of digital technologies and conduct large-scale, multicenter randomized controlled trials to longitudinally validate the specific mechanisms of different exercise models, thereby advancing the development of personalized, precision intervention protocols.

**Systematic review registration:**

Open Science Framework with the registration number (10.17605/OSF.IO/WYSJF).

## 1 Introduction

Cognitive frailty, a common geriatric syndrome, has gained increasing attention in aging research. It is defined as the simultaneous presence of cognitive frailty and physical frailty, excluding Alzheimer’s disease and other forms of dementia (1). The concept of cognitive frailty has shifted the research perspective from a unidimensional focus on older adults’ cognitive or physical functions to a multidimensional assessment of age-related declines in physical health, cognition, and functional capacity. This provides a comprehensive perspective for understanding vulnerability in later life, holding significant implications for promoting healthy aging. As the global aging process accelerates, the prevalence rates of cognitive frailty are also increasing markedly (2). Studies indicate that the prevalence of cognitive frailty ranges from 4.4 to 39.7% (3). Furthermore, cognitive frailty increases the risk of adverse health outcomes, including falls, disability, prolonged hospitalization, and even mortality (4). This severely impacts the quality of life and physical and mental health of older adults while imposing substantial care burdens and economic pressures on healthcare systems (5).

Notably, cognitive frailty is a dynamic and reversible condition (6). Early identification and effective intervention may help delay its progression and reduce negative health outcomes. Among non-pharmacological approaches, exercise interventions are recognized as promising due to their cost-effectiveness and accessibility (7). Research indicates that exercise interventions can enhance cognitive function in older adults by reducing oxidative stress and inflammatory marker activity, as well as promoting synaptic plasticity (8). Recent meta-analyses (9, 10) have demonstrated that exercise interventions significantly improve physical function, such as mobility, muscle strength, and balance, in older adults with cognitive frailty, while also enhancing overall cognitive performance and quality of life. These benefits may be associated with synergistic effects involving improved cerebral blood flow perfusion and the release of neurotrophic factors.

Despite the effectiveness of exercise interventions, their traditional implementation models often pose challenges for long-term adherence due to high costs associated with frequent patient visits and low compliance with self-management. Digital exercise, however, leverages technologies such as wearable devices, mobile applications, and artificial intelligence to enable real-time monitoring of patient exercise, personalized guidance, and remote supervision (11), offering a novel solution to this problem. Previous research reviews have demonstrated that digital exercise interventions exhibit unique advantages in overcoming spatial and temporal constraints and enhancing training motivation in fields such as stroke rehabilitation (12) and joint disease management (13). However, there is currently no scoping review that systematically evaluates the effectiveness of digital exercise for cognitive frailty, nor has there been a comparative and integrative analysis of traditional versus digital exercise in this population. Furthermore, evidence regarding the biological mechanisms through which exercise improves cognitive frailty remains inadequately synthesized.

Given this, to integrate fragmented evidence, this study innovatively adopts a multidimensional integrated analysis framework centered on “exercise formats-mechanisms-health outcomes.” This approach provides a clear, structured perspective for understanding the intervention effects of different exercise programs on cognitive frailty, elucidating their underlying biological mechanisms, and exploring their enabling pathways and implementation possibilities from a new digital exercise perspective. Consequently, it offers valuable reference and insights for designing personalized, mechanism-driven intervention programs.

## 2 Materials and methods

This review followed the Joanna Briggs Institute (JBI) methodological framework for scoping reviews (14) and was reported in accordance with the PRISMA-ScR guidelines (15). The study protocol was registered on the Open Science Framework with the registration number (10.17605/OSF.IO/WYSJF).

### 2.1 Research questions

The study encompasses the following questions: (1) What exercise intervention programs are currently applied to improve and delay cognitive frailty in older adults, and what are their specific contents? (2) Are exercise intervention programs effective? (3) Does the literature propose potential biological mechanisms underlying the effects of exercise interventions on cognitive frailty?

### 2.2 Literature search

The literature search was conducted using PubMed, Cochrane Library, Embase, Web of Science, and Scopus databases. All relevant articles published from the inception of each database up to October 9, 2025, were searched using a combination of Medical Subject Headings (MeSH) terms and free-text keywords. To ensure comprehensive retrieval, additional studies were identified through cross-referencing within. Key concepts in the search include “exercise,” “cognitive frailty,” “frail older adult,” and “cognitive dysfunction.” Detailed search terms, concepts, and strategies are provided in Supplementary Appendix 1.

### 2.3 Inclusion and exclusion criteria

Inclusion criteria: (1) Participants aged ≥60 years diagnosed with or defined as having cognitive frailty. (2) Study designs: randomized controlled trials, quantitative studies, or mixed-methods studies. (3) Interventions comprised exercise interventions for older adults with cognitive frailty or combined interventions integrating exercise.

Exclusion criteria: (1) Studies involving participants exhibiting only frailty or cognitive impairment, or those with dementia or psychiatric disorders. (2) Literature types such as reviews, animal studies, protocols, conference abstracts or posters, and studies with inaccessible full texts. (3) Literature lacking detailed descriptions of exercise intervention content or outcome measures, or intervention protocols including confounding factors such as medication or physical therapy.

### 2.4 Literature screening

EndNote X9 was used to manage and screen retrieved literature. After removing duplicates, two trained researchers independently conducted an initial screening based on inclusion and exclusion criteria by reviewing titles and abstracts. Subsequently, they independently reviewed the full text for further screening. Any discrepancies were resolved through discussion with a third researcher until a consensus was reached.

### 2.5 Data extraction and synthesis

Data were extracted from the included studies, covering the following: author, publication year, country, study design, diagnostic criteria for cognitive frailty, sample size, types of exercise intervention, and outcome measures. To further detail the exercise interventions, we also extracted specific parameters of the exercise protocols, including intervention venue, duration, frequency, intensity, and cycle. Additionally, we extracted data on potential mechanisms underlying the effects of exercise interventions on cognitive frailty. The first author created the data extraction form, which was reviewed and supplemented by the second author. Any discrepancies between the authors were resolved through discussion and consultation with a third researcher to ensure accuracy and consistency in the data extraction process.

### 2.6 Methodological considerations

To ensure consistency in the concept of cognitive frailty, all included studies adopted the definition of cognitive frailty in older adults proposed by the International Association for Nutrition and Aging (IANA) and the International Association of Gerontology and Geriatrics (IAGG) (1). However, due to the absence of a unified assessment tool for cognitive frailty, diagnostic criteria may vary across studies. As this study is a scoping review aimed at mapping and summarizing the effects of exercise interventions on cognitive frailty—rather than quantifying or eliminating statistical heterogeneity—such variations in diagnostic criteria reflect the conceptual and methodological diversity within the field. To ensure transparency, details regarding study design, diagnostic criteria, and sample characteristics are summarized in the results section.

### 2.7 Quality assessment

Two researchers independently applied the Mixed Method Appraisal Tool (MMAT, 2018 version) (16) to assess the quality of included studies. This involved answering two screening questions and five criteria per study type (qualitative studies, quantitative randomized controlled trials, quantitative non-randomized studies, quantitative descriptive studies, mixed-method studies). After completion, evaluators cross-checked results, resolving any disagreements through discussion with a third researcher.

## 3 Results

### 3.1 Search results

**Figure 1:**
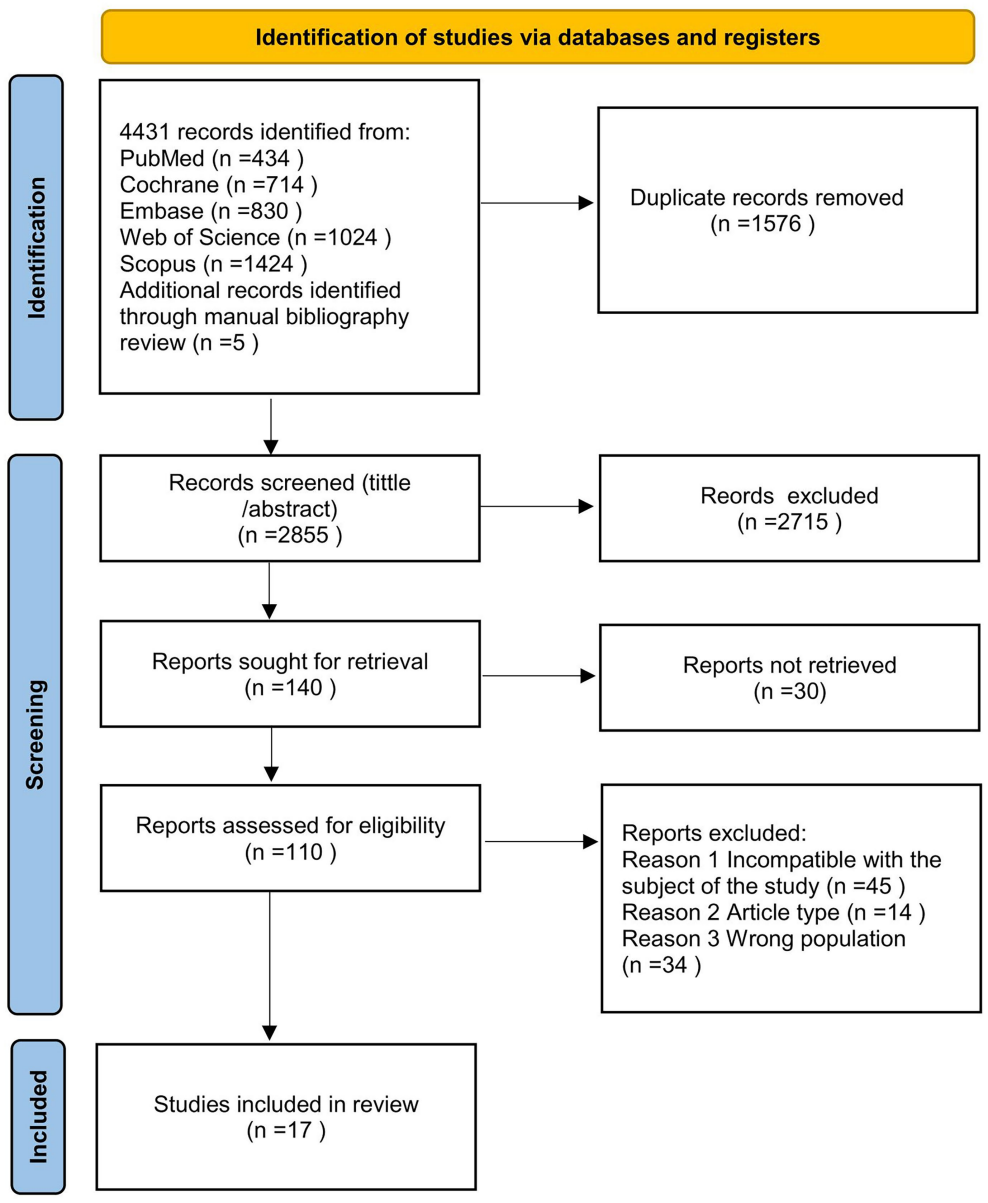
PRISMA 2020 flow diagram for new systematic reviews which included searches of databases and registers only. Consider, if feasible to do so, reporting the number of records identified from each database or register searched (rather than the total number across all databases/registers). If automation tools were used, indicate how many records were excluded by a human and how many were excluded by automation tools.

The initial search yielded 4,431 records. After duplicate removal, 1,576 articles were excluded, leaving 2,855. After screening titles and abstracts, 2,715 articles were excluded, leaving 140 documents. Following a secondary screening, 30 articles with unavailable full texts were excluded, resulting in 110 remaining documents. After further full-text evaluation, 45 articles were excluded for non-compliance with the research theme, 14 for mismatched literature types, and 34 for inappropriate study populations. Ultimately, 17 studies met the inclusion criteria (Figure 1).

### 3.2 Study characteristics

This scoping review included 17 studies published between 2018 and 2025 from four countries: China (*n* = 14), the United States (*n* = 1), South Korea (*n* = 1), and Canada (*n* = 1). The included studies comprised 16 randomized controlled trials and one quasi-experimental study. Tables 1, 2 presents the key details of the included studies.

**Table 1:** Basic characteristics of the study (*N* = 17).

Author/year	Country	Study type	Diagnostic criteria of CF	Participants		Interventions		Interventions model	Outcomes
				Age (years, I/C)	Sample size (I/C)	Intervention group	Control group		
Kwan et al. (2020) (17)	China	RCT	FP ≥ 1 point; MoCA < 25 points; CDR = 0.5	I:70.5 (IQR7.0) C:71.0 (IQR14)	I:16 C:17	Aerobic exercise	Conventional Behavior Change Intervention	Digital exercise (Samsung Health and WhatsApp)	A① B④⑥
Yoon et al. (2018) (18)	Korea	RCT	FP ≥ 1 point; CDR = 0.5	I:73.82 ± 4.37 C:74.03 ± 4.27	I:20 C:23	Resistance training	Balance and band stretching	Traditional exercise	A④ B①②③④
Wu et al. (2025) (19)	China	RCT	FP ≥ 1 point; MoCA < 26 points	I:70.12 ± 0.96 C:68.18 ± 0.98	I:34 C:34	Resistance training	Senior fitness exercise	Digital exercise (Tencent Meeting)	A①②⑤ B①④⑤ C① D①
Liu et al. (2018) (20)	USA	RCT	SOF ≥ 1point; 3MSE < 88 points	I:78.6 ± 5.2 C:79.1 ± 5.3	I:262 C:290	Multi-component exercise	Health education	Traditional exercise	A③ B①
Ye et al. (2021) (21)	China	RCT	FP ≥ 3 points; Diagnosed with mild cognitive impairment, according to the Petersen criteria	I:72.33 ± 6.21 C:72.92 ± 7.19	I:45 C:45	Multi-component exercise	Health education	Traditional exercise	A① B④⑨
Chen et al. (2021) (22)	China	RCT	FP ≥ 1 point; MoCA (BeijingVersion) score of 19–25;	I:84.59 ± 4.21 C:84.75 ± 5.41	I:29 C:30	Otago exercise	Health education	Traditional exercise	A⑥ B② C① D②
Falck et al. (2025) (23)	Canada	RCT	SPPB ≤ 9points; MoCA < 26 points	I:81.62 ± 6.55 C:83.31 ± 5.88	I:93 C:99	Otago exercise	Usual care	Traditional exercise	A① B①⑧
Zhu et al. (2023) (24)	China	quasi-experimental study	FP ≥ 1 point; CDR = 0.5	I:72.66 ± 6.54 C:72.88 ± 5.78	I:35 C:34	Exergaming	Usual care	Digital exercise (HappyGoGo software)	A① C②
Jia et al. (2022) (25)	China	RCT	FP ≥ 1 point; CDR = 0.5	I:71.3 ± 5.0 C:70.8 ± 4.2	I:30 C:30	Mindfulness Tai-Chi Chuan	Mindfulness intervention	Traditional exercise	A② B①②⑦
Wan et al. (2022) (26)	China	RCT	EFS ≥ 5 points; MoCA (FuzhouVersion) ≤ 26 points; GDS score of 2–3	I:67.31 ± 5.58 C:64.71 ± 5.07	I:26 C:24	Baduanjin exercise	Usual care	Traditional exercise	A①
Lin et al. (2023) (27)	China	RCT	EFS ≥ 5 points; MoCA ≤ 2 points; GDS score of 2–3	I:67.68 ± 5.19 C:65.35 ± 5.15	I:51 C:51	Baduanjin exercise	Usual care	Traditional exercise	A①
Ye et al. (2024) (28)	China	RCT	Physical frailty; More than 1.5 standard deviation below the mean for age-, gender-, and education-adjusted norms on any cognitive function test; No dependency in instrumental activities of daily living	I:67.68 ± 5.19 C:65.35 ± 5.15	I:51 C:51	Baduanjin exercise	Usual care	Traditional exercise	A①
Wang et al. (2024) (29)	China	RCT	EFS ≥ 5 points; MoCA ≤ 26 points; GDS score of 2–3	I:66.90 ± 4.54 C:67.64 ± 5.49	I:60 C:60	Baduanjin exercise	Usual care	Traditional exercise	A①⑤⑦⑧⑨
Yang et al. (2023) (30)	China	RCT	FP ≥ 1 point; MoCA (BeijingVersion) < 25 points	I:67.5 ± 3.1 C:67.4 ± 2.8	I:40 C:39	Baduanjin exercise combined with cognitive training	Usual care	Digital exercise (“With Sugar”APP platform)	A① B③④
Kwan et al. (2021) (31)	China	RCT	FP ≥ 1 point; MoCA < 25 point; CDR = 0.5	I:73.0(IQR7.5) C:77.5(IQR15.3)	I:9 C:8	Virtual reality motor-cognitive training	Non-VR sequential motor-cognitive training	Digital exercise (VR platform)	A① B②④
Lai et al. (2025) (32)	China	RCT	FP ≥ 3 points; MoCA < 26 points; CDR = 0.5	I:74.81 ± 8.23 C:76.50 ± 7.75	I:36 C:36	Exercise-cognitive dual-task training	Health education	Digital exercise (WeChat application)	A① B②
Liao et al. (2025) (33)	China	RCT	FP ≥ 1 point; MMSE≥24points; MoCA <26 points	I:77.5 ± 5.8 C:79.3 ± 6.9	I:20 C:19	Boxing-cycling dual-task training	Stationary cycling	Digital exercise (wireless inertial sensors)	A①⑤

I: Intervention group; C: Control group; FP: Fried Phenotype; EFS: Edmonton Frail Scale; SOF: the Study of Osteoporotic Fractures frailty index; CDR: Clinical Dementia Rating; GDS: Global Deterioration Scale. A for cognitive function: ① MoCA: Montreal Cognitive Assessment; ② MMSE: Mini-mental State Examination; ③ MMSE: Modified Mini-Mental State Examination; ④ FAB: Frontal Assessment Battery; ⑤ TMT-A and TMT-B: Trail Making Test parts A and B; ⑥ FTSST: Five Times Sitto Stand Test; ⑦ Clock Drawing Test; ⑧ Verbal Fluency Test; ⑨ Stroop Test. B for physical function: ① SPPB: Short Physical Performance Battery; ② TUG: Timed Up and Go Test; ③ Gait speed; ④ Grip strength; ⑤ 4MWP: 4-meter walk speed; ⑥ 6MWT: six-minute walk test; ⑦ the 30-s Chair test; ⑧ the rate of falls; ⑨ 6MWT: 6-meter walking time. C for psychological state: ① GDS: Geriatric Depression Scale; ② Loneliness Scale. D for quality of life: ① QoL-AD: Quality of Life in Alzheimer’s Disease scale; ② SF-12:12 Item Short Form Health Survey.

**Table 2:** Basic characteristics of exercise intervention programs.

Author/year	Types of exercise intervention	Interventions model	Intervention venue	Intervention content	Duration	Frequency	Intensity	Cycle
Kwan et al. (2020) (17)	Aerobic exercise	Digital exercise (Samsung Health and WhatsApp)	Community centers	The first 2 weeks: brisk walking training; 3 to 12 weeks: self-paced brisk walking training + Mobile health interventions	The walking duration was adjusted based on the participants’ initial fitness levels and their ongoing progress.	Prefrail:5–10 times /week, Frail: 3–5 times /week	N/A	12 weeks
Yoon et al. (2018) (18)	Resistance training	Traditional exercise	Community centers	10 min warm-up,40 min high-speed resistance training and 10 min of cooling down;	60 min/time	3 times /week	RPE12-13	16 weeks
Wu et al. (2025) (19)	Resistance training	Digital exercise (Tencent Meeting)	1–6 weeks: Offline group training; 7–12 weeks: Online training via Tencent Meeting	5 min warm-up, 20–40 min of exercise (20 min in the initial, 30 min in mid-term, 40 min in final phase), and a 5-min cool down	30–50 min/time	3 times /week;	RPE10-14	12 weeks
Liu et al. (2018) (20)	Multi-component exercise	Traditional exercise	Senior Activity Centre	30 min walking,10 min lower body strength training,3–5 min flexibility and 10 min balance	53-55 min/time	2 times /week;	Walking: RPE 13; lower body strength training: RPE 15–16	24 months
Ye et al. (2021) (21)	Multi-component exercise	Traditional exercise	Community health service center or family	5 min aerobic exercise, 20 min resistance exercise, 10 min balance training, and 10 min flexibility training	45 min/time,	3 times /week	RPE 12–14	12 weeks
Chen et al. (2021) (22)	Otago exercise	Traditional exercise	Nursing home	5 min warm-up, 10 min resistance training and 15 min balance exercise.	30 min/time	3 times /week	N/A	12 weeks
Falck et al. (2025) (23)	Otago exercise	Traditional exercise	Home-based exercise	The Otago exercise program includes 5 strengthening exercises and 11 balance retraining exercises.	30 min/time	2 times /week	N/A	12 months
Zhu et al. (2023) (24)	Exergaming	Digital exercise (HappyGoGo software)	Community centers	5 min warm-up, 30 min exergaming and 5 min cool-down	40 min/time	2 times /week	N/A	8 weeks
Jia et al. (2022) (25)	Mindfulness Tai-Chi Chuan	Traditional exercise	First 3 months: at senior or community centers; Final 3 months: individual practice	Mindfulness: It consisted of four basic forms of meditation practices (body scan, walking meditation, gentle yoga, sitting meditation). Tai-Chi Chuan:10 min warm-up, 45 min exercises and 5 min cool-down	60 min/time,	2 times /week	N/A	6 months
Wan et al. (2022) (26)	Baduanjin exercise	Traditional exercise	Community centers	15 min warm-up, 40 min Baduanjin training and 5 min cool down	60 min/time	3 times /week	N/A	24 weeks
Lin et al. (2023) (27)	Baduanjin exercise	Traditional exercise	Community centers	15 min warm-up, 40 min Baduanjin training and 5 min cool down	60 min/time	3 times /week	N/A	24 weeks
Ye et al. (2024) (28)	Baduanjin exercise	Traditional exercise	Community centers	15 min warm-up, 40 min Baduanjin training and 5 min cool down	60 min/time	3 times /week	N/A	24 weeks
Wang et al. (2024) (29)	Baduanjin exercise	Traditional exercise	Community centers	15 min warm-up, 40 min Baduanjin training and 5 min cool down	60 min/time	3 times /week	N/A	24 weeks
Yang et al. (2023) (30)	Baduanjin exercise combined with cognitive training	Digital exercise (“With Sugar”APP platform)	Hospital and home online check-in	Baduanjin exercise: 15 min warm-up, 40 min Baduanjin training and 5 min cool down. Cognitive training: including finger exercises, image recognition and recall, attention training, poker classification and sorting, anti reverse practice.	60 min/time	3 times /week	The intensity of exercise is evaluated based on the percentage of maximum heart rate, which is equal to [average heart rate/(220-age)] × 100%	12 weeks
Kwan et al. (2021) (31)	Virtual reality motor-cognitive training	Digital exercise (VR platform)	Older adult community center	Cognitive training: including orientation, find a bus stop, reporting lost items, find a supermarket, grocery shop, cook, find a travel hotspot, and bird watch. Motor training: cycling on an ergometer	30 min/time	2 times /week	N/A	8 weeks
Lai et al. (2025) (32)	Exercise-cognitive dual-task training	Digital exercise (WeChat application)	The first 2 weeks: at the community health center; 3 to16 weeks: home-based	5–10 min warm-up, 40 min exercise-cognitive dual-task training and 5–10 min cool down.	50–60 min/time	3 times /week;	Intensity: Training intensity was determined by heart rate, with the appropriate range calculated as (220-age) × (60–80%)	16 weeks
Liao et al. (2025) (33)	Boxing-cycling dual-task training	Digital exercise (wireless inertial sensors)	Older adult apartments and daycare facilities	Participants engaged in an interactive boxing-cycling session on a stationary bike fitted with an interactive boxing panel. During interactive boxing-cycling, participants aimed to strike targets with precision and speed, while also maintaining a pedaling speed of 60 RPM.	40 min/time	3 times /week	RPE 14–16	12 weeks

### 3.3 Quality assessment

The included studies were generally of high quality, with one study rated as moderate quality. See Supplementary Appendix 1 for details. The overall evidence demonstrated good consistency and robustness, which did not affect the systematic integration of exercise intervention effects and mechanisms in this study.

### 3.4 Exercise interventions for cognitive frailty

#### 3.4.1 Types of exercise interventions

The 17 studies included in this review demonstrate that exercise intervention programs primarily consisted of aerobic exercise (17), resistance training (18, 19), multicomponent exercise (20–24), traditional fitness qigong exercises (25–30), and dual-task training (30–33). In terms of delivery mode, interventions were categorized as either traditional in-person (18, 20–23, 25–29) or digitally supported (17, 19, 24, 30–33). Regarding the distribution of exercise types, six studies employed traditional fitness qigong: one focused on Tai Chi (25) and five on Baduanjin (26–30). Four studies involved multi-component exercise: two combined aerobic exercise, resistance training, balance training, and flexibility training (20, 21); two used Otago Exercise combining resistance training and balance training (22, 23); and one employed exercise games integrating resistance and balance training (24). Four studies reported dual-task training, including Baduanjin combined with cognitive training (30), virtual reality motor–cognitive training (31), exercise–cognitive dual-task training (32), and boxing–cycling dual-task training (33). Notably, nearly half of the included studies adopted digitally delivered interventions, highlighting the potential and value of digital tools as effective intervention in this field.

#### 3.4.2 Duration, frequency, intensity, and duration of exercise interventions

Across different studies, variations exist in the settings, frequency, intensity, and duration of exercise interventions. (1) Intervention settings: Traditional exercise interventions primarily rely on community centers (17, 18, 24, 26–29), senior activity centers (20, 31), and nursing homes (22, 33) to ensure standardized movement instruction and promote social interaction among participants. Digital exercise interventions, however, predominantly adopt a hybrid model combining initial in-person training with subsequent online home-based check-ins (19, 30, 32), reflecting the design advantages of accessibility and long-term monitoring. (2) Exercise Frequency: Included studies predominantly employed exercise frequencies of twice weekly (20, 23–25, 31) or thrice weekly (18, 19, 21, 22, 26–30, 32, 33), with intervention duration varying according to specific exercise program designs. (3) Exercise Intensity: Traditional resistance training (18, 19) and multi-component exercise (20, 21) tended to use the Borg Rate of Perceived Exertion scale to set exercise intensity, while digitally supported dual-task training (30, 32) primarily referenced heart rate during exercise to assess and adjust participants’ exercise intensity. (4) Duration: The intervention periods in the included studies varied considerably, ranging from a minimum of 8 weeks (24, 31) to a maximum of 24 months (20). The most common durations were 12 weeks (17, 19, 21, 22, 30, 33) and 24 weeks (26–29).

#### 3.4.3 Characteristics of digital exercise interventions

Research indicates that digital exercise interventions improve cognitive frailty primarily through two pathways: remote supervision and guidance, and immersive game interactions. Four studies mentioned overcoming geographical constraints via online meetings (19) or applications (17, 30, 33) to enable remote supervision and management. Three studies (24, 31, 33) utilized digital exercise games to enhance exercise enjoyment and participation motivation.

This shift from traditional offline interventions to an integrated online-offline model not only expands the scalability of interventions but also creates opportunities to further enhance their public health benefits. (1) Safety: Four studies, respectively, employed wrist-worn step counter (17), wrist-worn heart rate sensor (31), validated chest-strap heart-rate monitor (32), wireless inertial sensors (33) to record kinematic data such as speed, cadence, and stride length. These devices monitored participants’ heart rates and exercise tolerance to enable real-time adjustment of exercise intensity, ensuring exercise safety. Additionally, studies indicated (31) no adverse outcomes related to VR sickness occurred during the research period. (2) Adherence: Digital exercise programs adopted multiple strategies to promote adherence, including praise and rewards (17), online training (19), virtual reality (31), platform check-ins (30, 32), and exercise games (24, 31, 33). These approaches not only enhance motivation and enjoyment in older adults exercise but also meet the convenience needs for remote home supervision and management. (3) Personalization: Research findings (30, 32) indicate that tracking participants’ initial physical fitness levels and activity progress provides data support for delivering personalized exercise prescriptions.

### 3.5 Differences in diagnostic criteria for cognitive frailty

Currently, there are no unified diagnostic criteria for cognitive frailty. Researchers predominantly use a combination of physical frailty and cognitive function assessment scales to screen older adults for cognitive frailty. Among these, the most commonly employed combinations include: the Fried frailty phenotype (FP)combined with the Clinical Dementia Rating (CDR) scale (18, 24, 25), the FP combined with the Montreal Cognitive Assessment (MoCA) (19, 22, 30), the FP combined with both CDR and MoCA (17, 31, 32), and the Edmonton Frailty Scale (EFS) combined with MoCA and the Global Deterioration Scale (GDS) (26, 27, 29). However, even within the same combination, variations exist in the versions of the scales used and their respective scoring criteria.

### 3.6 Outcomes and effects of exercise interventions

#### 3.6.1 Effects of exercise intervention on cognitive function

Among the 17 included studies, cognitive function was the most widely assessed outcome measure. The MoCA was employed as the primary cognitive assessment tool in 13 studies (17, 19, 21, 23, 24, 26–33), while other instruments included the MMSE (19, 25), 3MSE (20), and TMT-A/B (19, 29, 33). Results indicated that most exercise interventions significantly improved overall cognitive function scores while positively impacting executive function, attention, and memory. However, despite being resistance training, findings on executive function varied across studies. Yoon et al. (18) reported significant improvements in FAB scores, whereas Wu et al. (19) found no significant enhancement in TMT-A/B test performance.

#### 3.6.2 Effects of exercise intervention on physical function

Eleven studies (17–23, 25, 30–32) reported the effects of exercise interventions on physical function. Notably, different exercise types exerted varying impacts on physical function. Resistance training (18, 19) primarily focused on positively influencing muscle strength, endurance, and activities of daily living; while Baduanjin (26–30) effectively enhances upper and lower limb gait and dynamic balance. In contrast, multi-component exercise programs, which integrate multiple exercise modalities, typically yield more pronounced combined effects than single-exercise interventions. They multidimensionally improve participants’ physical function (20), walking efficiency (21), and reduce fall risk (23).

#### 3.6.3 Effects of exercise interventions on psychosocial outcomes

Compared to previous domains, evidence regarding the impact of exercise on psychosocial outcomes is limited and inconclusive. Only two studies (19, 22) reported statistically significant improvements (*p* < 0.05) in quality of life scores among exercise intervention groups relative to control groups. Three studies (19, 22, 24) examined the effects of exercise interventions on psychological states. Chen et al. (22) demonstrated that exercise significantly reduced participants’ depression scores, whereas Wu et al. (19) found no statistically significant effect on depression. Additionally, Zhu et al. (24) observed that exercise game interventions did not significantly reduce loneliness among participants with cognitive frailty.

### 3.7 Potential biological mechanisms by which exercise interventions influence cognitive frailty

We also extracted and summarized the mechanisms by which exercise interventions influence cognitive frailty as described in the included studies. Seven studies briefly explained potential mechanisms, primarily encompassing five types. (1) Brain Structure and Plasticity: Exercise interventions delay volume atrophy in hippocampal subregions (left parasubiculum, left HATA, right CA1, and right presubiculum) (26), enhance functional connectivity within the prefrontal-hippocampal network, and improve cognitive function by modulating brain structural and functional plasticity. This effectively slows memory decline in older adults.(2) Prefrontal Activation and Neural Efficiency: Exercise intervention enhances prefrontal cortical activation levels (33), influencing neural efficiency to promote shorter reaction times and improved accuracy. (3) Neuroinflammation and Oxidative Stress: Exercise intervention regulates inflammatory cytokine levels (IFN-*γ*, IL-2, IL-4), suppresses inflammasome activation (28), enhances antioxidant enzyme activity (20), and reduces oxidative stress-induced neuronal damage. (4) Neurotransmitter changes: Exercise intervention enhances neuronal plasticity by upregulating brain-derived neurotrophic factor levels (21). (5) Improved Cerebral Blood Flow and Metabolism: Exercise increases blood flow parameters in key cerebral vessels, including peak systolic velocity (PSV), mean blood flow velocity (MBFV), and end-diastolic velocity (EDV) in the right middle cerebral artery, PSV in the left middle cerebral artery, and MBFV and EDV in the basilar artery (27), thereby enhancing cerebral oxygen supply. It also reduces glycated hemoglobin levels (30), contributing to delayed progression of cognitive frailty (Table 3).

**Table 3:** Mechanisms of exercise intervention in improving cognitive frailty.

Author/year	Types of exercise intervention	Mechanisms	Study results
Liu et al. (2018) (20)	Multi-component exercise	IL-6→	The study demonstrated that a 24-month structured, moderate-intensity physical activity program reduced the severity of cognitive frailty compared with a health education program among sedentary older persons and that this benefit was not modified by baseline IL-6.
Ye et al. (2021) (21)	Multi-component exercise	Albumin↑, prealbumin↑, Transferrin↑, lymphocyte count↑	Multi-component exercise may improve cognitive frailty by affecting the expression of some brain-derived neurotrophic factors, which can regulate brain plasticity and function.
Wan et al. (2022) (26)	Baduanjin exercise	Atrophy of hippocampal subregions ↓	Baduanjin exercise intervention slowed the atrophy of hippocampal subregions, including the left parasubiculum, left HATA, right CA1 and right presubiculum, suggesting that its effect on improving cognitive frailty may be related to the structural plasticity changes of the hippocampal subregion.
Lin et al. (2023) (27)	Baduanjin exercise	Cerebral blood flow perfusion↑	Baduanjin training had a positive effect on cerebral blood flow, increasing PSV, MBFV and EDV in right middle cerebral artery, PSV in left middle cerebral artery, and MBFV and EDV in basilar artery, suggesting possible mechanisms for improvement of cognitive and physical function.
Ye et al. (2024) (28)	Baduanjin exercise	MDA↓, 8-iso-PGF2α ↓, SOD ↑	Baduanjin exercise may improve physical frailty and cognitive function in community-dwelling older adults with cognitive frailty by modulating oxidative stress and inflammation, through reducing pro-oxidative markers levels (MDA, 8-iso-PGF2α), increasing the activity of antioxidant marker SOD, and regulating the levels of inflammatory cytokines (IFN-*γ*, IL-2, IL-4).
Yang et al. (2023) (30)	Baduanjin exercise combined with cognitive training	HbA1c↓	Baduanjin exercise combined cognitive training can slow down the decline of cognitive function in the aged patients with diabetes, improve their physical weakness, and reduce their blood sugar level.
Liao et al. (2025) (33)	Boxing-cycling dual-task training	HbO↓	The mechanism by which boxing-cycling dual-task training impacts cognitive frailty involves changes in prefrontal cortex activation. This training modality effectively reduces activation in the prefrontal cortex, thereby enhancing neural efficiency and improving cognitive function.

“↑”: Increased or improved; “↓”: Reduced or slowed; “→”: Irrelevant.

## 4 Discussion

This scoping review employs a multidimensional integration framework linking “exercise formats-mechanisms-health outcomes” to systematically reveal the synergistic network through which exercise interventions improve cognitive frailty. Results indicate that despite significant variations in exercise intervention types, diagnostic criteria, and outcome measures, exercise demonstrates substantial potential in enhancing cognitive function, physical health, overall quality of life, and mental health among older adults with cognitive frailty. However, current exercise interventions are constrained by inconsistent diagnostic criteria, insufficient long-term adherence, and fragmented intervention measures. Therefore, leveraging digital health technologies to explore personalized exercise dosing, real-time feedback, and sustainable supervision is crucial for achieving precision and individualization in the prevention and treatment of cognitive frailty.

### 4.1 Promote the diversification of intervention formats and enhance the sustainability of intervention measures

Regarding exercise intervention formats, they are primarily categorized into offline and blended online-offline approaches. Face-to-face offline interventions demonstrate significant advantages in real-time guidance and structured supervision, aiding in the prevention of fall risks (23). Furthermore, offline interventions maintain robust social interaction support, enhancing psychological belonging (22). However, limitations in accessibility and long-term monitoring may arise due to geographical and temporal constraints. Against this backdrop, digital technology—leveraging powerful exercise parameter analysis, real-time remote guidance capabilities, and immersive application scenarios—has deeply integrated into exercise interventions, offering a more effective and sustainable practice paradigm for improving cognitive frailty in older adults. Although digital exercise offers unique advantages in quantifying movement data, enhancing training motivation, and overcoming temporal and spatial constraints, barriers remain in widespread adoption and long-term efficacy validation. This stems from the fact that digital exercise programs require participants to engage in sustained self-management at home. In this context, the complexity of digital technology usage may pose learning and acceptance challenges for older adults (34), potentially exacerbating their feelings of loneliness (24).

Therefore, further efforts should be made to integrate user-friendly digital interfaces, simplify functional designs to meet the needs of diverse populations, and strengthen technology training for older adults, thereby optimizing the acceptability and adaptability of digital technology. Additionally, future efforts should fully integrate online and offline strengths: establishing trust foundations through offline components, then leveraging digital tools for long-term tracking and dynamic adjustments to enhance intervention accessibility and continuity. More importantly, real-time, multidimensional biofeedback systems must be strengthened. By leveraging an integrated framework of “exercise formats-mechanisms-health outcomes” dynamic models linking exercise parameters, mechanism pathways, and health outcomes should be developed. This will propel digital exercise toward a truly personalized, mechanism-driven intervention paradigm.

### 4.2 Systematically explore biological mechanisms to advance personalized interventions

The effectiveness of exercise interventions in improving cognitive frailty stems from their underlying biological mechanisms involving multi-pathway, multi-level synergistic effects. Aerobic exercise has been demonstrated to effectively enhance peak oxygen uptake, strengthen cardiopulmonary function, and consequently significantly increase cerebral blood flow while reducing central arterial stiffness (35), thereby promoting cognitive function improvement. Resistance training activates the bone-brain axis (36), stimulating skeletal muscle to release and upregulate irisin. This process elevates levels of IGF-1 and BDNF, thereby delaying skeletal muscle atrophy and promoting neuroplasticity and cognitive enhancement. Traditional fitness qigong exercises, as mind–body exercises integrating intention, breath, and movement, not only mitigate hippocampal subregion atrophy (26, 37) and but also induce neurogenesis and synaptic plasticity. Furthermore, it exerts neuroprotective effects by modulating oxidative stress and inflammation through reducing pro-oxidative markers (MDA, 8-iso-PGF2α), enhancing antioxidant enzyme SOD activity, and regulating inflammatory cytokine expression (IFN-*γ*, IL-2, IL-4) (28). Dual-task training simultaneously influences peripheral metabolism and central nervous system efficiency: peripherally, it reduces HbA1c levels (30) and alleviates metabolic stress; centrally, it enhances neural information processing efficiency and improves cognitive function by decreasing oxygenated hemoglobin concentration and activation levels in the prefrontal cortex (33). Multicomponent exercise, integrating aerobic exercise, resistance training, and balance training, further generates synergistic benefits across neural (21), metabolic (22), and immune (38) systems.

Collectively, these mechanisms illustrate that exercise interventions improve cognitive frailty not through a single pathway, but via the coordinated regulation of multiple, interconnected biological systems. These pathways span various dimensions, including neural plasticity, modulation of inflammation and oxidative stress, optimization of cerebral blood flow and metabolism, and neurotrophic support, forming a systemic adaptive network characterized by “peripheral–central–behavioral” integration. However, the literature included in this review offers limited mechanistic exploration. Only a portion of studies employed biomarker assessments, and objective neuroimaging evidence remains generally lacking. Future research must strengthen neuroimaging and biomarker analyses to identify potential biological targets for personalized interventions.

### 4.3 Optimize exercise intervention programs to promote comprehensive improvements in health outcomes

The studies included in this review indicate that exercise interventions for cognitively frail older adults encompass diverse approaches. Due to variations in intervention content and delivery, the effectiveness of exercise interventions in improving cognitive frailty among older adults also differs. Even among studies employing identical exercise programs, this review observed divergent intervention outcomes. Yoon et al. (18) demonstrated that 16 weeks of moderate-intensity high-speed resistance training significantly improved participants’ processing speed and executive function. Conversely, Wu et al. (19) found no significant effect on executive function, potentially due to the low intensity and short duration of their intervention, which may have been insufficient to activate the PI3K-AKT–mTOR pathway, thereby limiting adaptive remodeling in muscle and neural cells (39). In contrast, dual-task training (30–33)—by incorporating detailed motor parameters and simultaneously engaging peripheral and central systems—not only enhances coordination between physical activity and cognitive tasks but also accelerates cognitive switching speed, thereby boosting the brain’s adaptability and flexibility. Traditional fitness qigong exercises (25–30), as the primary exercise type for improving cognitive frailty, demonstrated more significant simultaneous improvements in both physical and mental health compared to single-modality aerobic exercise (17) and resistance training (18, 19). This may be attributed to its emphasis on the holistic coordination mechanism of “regulating the body, regulating the breath, and regulating the mind,” which integrates functional restoration with psychological regulation. Consequently, it demonstrates unique intervention advantages across multidimensional health outcomes.

Although these findings align with existing research (40), supporting the positive association between exercise dosage, intensity, and pattern diversity with cognitive function. However, our review reveals significant variability in the design of current exercise intervention parameters—including intensity, frequency, and duration—lacking systematicity and comprehensiveness. This limitation constrains the potential for greater improvement in cognitive frailty. Therefore, future exercise intervention designs should thoroughly explore optimal training ratios and progression models within consistent intervention frameworks. This approach will enable the optimization of exercise content and load, thereby promoting comprehensive improvements in patient health outcomes.

### 4.4 Limitations and future directions

Despite highlighting the positive role of exercise interventions in improving cognitive frailty, this review also acknowledges several limitations in the current evidence. First, most of the included studies were conducted in China, with limited representation from other regions, and generally featured small sample sizes, which may constrain the generalizability of the results. Therefore, future research should employ cross-cultural, multicenter, large-scale randomized controlled trials to validate the feasibility and applicability of different intervention protocols.

Second, the studies included in the review employed diverse diagnostic criteria and measurement tools. Although all reported significant improvements in cognitive frailty following exercise interventions, there was no uniformity in the target populations or settings. Furthermore, insufficient research exists on mental health outcomes, thereby undermining the standardization and comparability of findings. Future efforts should establish standardized diagnostic and assessment systems. This should involve integrating objective criteria to unify evaluation tools and applicable standards across different research settings, while strengthening the measurement of multifaceted health outcomes. Such measures will ensure the comparability, applicability, and practical utility of research findings.

Third, existing studies predominantly employ screening scales as primary outcome measures, with only a few incorporating biomarkers for corroboration, yet evidence remains limited. Future efforts should focus on developing a multidimensional, integrated assessment framework that organically combines functional screening, biomarkers, and multimodal neuroimaging to comprehensively elucidate intervention effects and underlying mechanisms.

Moreover, the integration of digital technology into exercise interventions remains relatively underdeveloped. Moving forward, we should fully leverage the potential of digital tools, relying on multidimensional bioinformation integration and real-time feedback technologies to continuously analyze and model participants’ exercise data. This will enable dynamic optimization of exercise programs, delivering precision and personalization in interventions.

## 5 Conclusion

This scoping review integrates the multidimensional “exercise formats-mechanisms-health outcomes” framework to comprehensively elucidate the efficacy and mechanistic evidence of exercise interventions in improving cognitive frailty. Results indicate that despite variations in exercise intervention models, diverse forms of exercise demonstrate significant potential in enhancing physical function, cognitive performance, and quality of life among older adults with cognitive frailty. This potential is evident not only in traditional exercise formats but is further extended through the integration of digital technologies such as virtual reality and mobile health, offering novel pathways for intervention. Simultaneously, we identified that diverse exercise modalities can improve cognitive frailty in older adults through multi-level biological mechanisms, characterized by multi-system coordination and spatiotemporal dynamic coupling. In summary, by critically synthesizing the existing evidence on the impact of exercise interventions on cognitive frailty, this review provides systematic insights to advance the field. Future research should be grounded in digital health technologies and employ multicenter, large-sample randomized controlled trials to verify the long-term effects and core mechanisms of exercise interventions.

## Data Availability

The original contributions presented in the study are included in the article/Supplementary material, further inquiries can be directed to the corresponding author.
