# Supplementary material for: The impact of exercise interventions on cognitive frailty: a scoping review of outcomes and biological mechanisms
Source: Front Public Health. 2026 Jan 12;13:1738522. doi: 10.3389/fpubh.2025.1738522 (PMC12832520; doi:10.3389/fpubh.2025.1738522)
Supplement: Supplementary file 1 [file Table_1.docx]

Supplementary Material

# Search strategy

Pubmed

| #1 | "exercise"[MeSH Terms] OR "acute exercise*"[Title/Abstract] OR "aerobic exercise*"[Title/Abstract] OR "aerobic training*"[Title/Abstract] OR "exercise training*"[Title/Abstract] OR "exercise therapy*"[Title/Abstract] OR "progressive resistance training*"[Title/Abstract] OR "progressive exercise training*"[Title/Abstract] OR "resistance training*"[Title/Abstract] OR "resistance exercise*"[Title/Abstract] OR "physical fitness"[Title/Abstract] OR "physical activit*"[Title/Abstract] OR "physical exercise*"[Title/Abstract] |
| --- | --- |
| #2 | "frail older adult"[MeSH Terms] OR "frail*"[Title/Abstract] OR "pre frail*"[Title/Abstract] OR "frailty syndrome"[Title/Abstract] OR "frailty"[Title/Abstract] OR "debility*"[Title/Abstract] |
| #3 | "cognitive dysfunction"[MeSH Terms] OR "cognitive frailty"[Title/Abstract] OR "cognitive decline"[Title/Abstract] OR "cognitive defect"[Title/Abstract] OR "cognition disorder"[Title/Abstract] OR "cognitive impairment"[Title/Abstract] OR "mental deterioration"[Title/Abstract] |
| #4 | #2 AND #3 |
| #5 | #1 AND #4 |

Web of science

| #1 | TS=("exercise" OR "acute exercise*" OR "aerobic exercise*" OR "aerobic training*" OR "exercise training*" OR "exercise therapy*" OR "progressive resistance training*" OR "progressive exercise training*"OR" resistance training*" OR "resistance exercise*" OR "physical fitness" OR "physical activit*" OR "physical exercise*") |
| --- | --- |
| #2 | TS=("frail older adult" OR "frail*" OR "pre frail*" OR "frailty syndrome" OR "frailty" OR "debility*" ) |
| #3 | TS=("cognitive dysfunction" OR "cognitive frailty" OR "cognitive decline" OR "cognitive defect" OR "cognition disorder" OR "cognitive impairment" OR "mental deterioration") |
| #4 | #2 AND #3 |
| #5 | #1 AND #4 |

Cochrane

| #1 | (exercise):ti,ab,kw OR (acute exercise*):ti,ab,kw OR (aerobic exercise*):ti,ab,kw OR (aerobic training*):ti,ab,kw OR (exercise training*):ti,ab,kw OR (exercise therapy*):ti,ab,kw OR (progressive resistance training*):ti,ab,kw OR (progressive exercise training*):ti,ab,kw OR (resistance training*):ti,ab,kw OR (resistance exercise*):ti,ab,kw OR (physical fitness):ti,ab,kw OR (physical activit*):ti,ab,kw OR (physical exercise*):ti,ab,kw |
| --- | --- |
| #2 | (frail older adult):ti,ab,kw OR (frail*):ti,ab,kw OR (pre frail*):ti,ab,kw OR (frailty syndrome):ti,ab,kw OR (frailty):ti,ab,kw OR (debility*):ti,ab,kw |
| #3 | (cognitive dysfunction):ti,ab,kw OR (cognitive frailty):ti,ab,kw OR (cognitive decline):ti,ab,kw OR (cognitive defect):ti,ab,kw OR (cognition disorder):ti,ab,kw OR (cognitive impairment):ti,ab,kw OR(mental deterioration):ti,ab,kw |
| #4 | #2 AND #3 |
| #5 | #1 AND #4 |

Scopus

| #1 | TITLE-ABS-KEY ("exercise" OR "acute exercise*" OR "aerobic exercise*" OR "aerobic training*" OR "exercise training*" OR "exercise therapy*" OR "progressive resistance training*" OR "progressive exercise training*" OR "resistance training*" OR "resistance exercise*" OR "physical fitness" OR "physical activit*" OR "physical exercise*") |
| --- | --- |
| #2 | TITLE-ABS-KEY ("frail older adult" OR "frail*" OR "pre frail*" OR "frailty syndrome" OR "frailty" OR "debility*" ) |
| #3 | TITLE-ABS-KEY ("cognitive dysfunction" OR "cognitive frailty" OR "cognitive decline" OR "cognitive defect" OR "cognition disorder" OR "cognitive impairment" OR "mental deterioration") |
| #4 | #2 AND #3 |
| #5 | #1 AND #4 |

Embase

| #1 | 'exercise':ti,ab,kw OR 'acute exercise*':ti,ab,kw OR 'aerobic exercise*':ti,ab,kw OR 'aerobic training*':ti,ab,kw OR 'exercise training*':ti,ab,kw OR 'exercise therapy*':ti,ab,kw OR 'progressive resistance training*':ti,ab,kw OR 'progressive exercise training*':ti,ab,kw OR 'resistance training*':ti,ab,kw OR 'resistance exercise*':ti,ab,kw OR 'physical fitness':ti,ab,kw OR 'physical activit*':ti,ab,kw OR 'physical exercise*':ti,ab,kw |
| --- | --- |
| #2 | 'frail older adult':ti,ab,kw OR 'frail*':ti,ab,kw OR 'pre frail*':ti,ab,kw OR 'frailty syndrome':ti,ab,kw OR 'frailty':ti,ab,kw OR 'debility*':ti,ab,kw |
| #3 | 'cognitive dysfunction':ti,ab,kw OR 'cognitive frailty':ti,ab,kw OR 'cognitive decline':ti,ab,kw OR 'cognitive defect':ti,ab,kw OR 'cognition disorder':ti,ab,kw OR 'cognitive impairment':ti,ab,kw OR 'mental deterioration':ti,ab,kw |
| #4 | #2 AND #3 |
| #5 | #1 AND #4 |

# Supplementary Tables

MMAT quality assessment tool

| **Category of study designs** | **Methodological quality criteria** | Study | | | | | | | |
| --- | --- | --- | --- | --- | --- | --- | --- | --- | --- |
|  |  | Kwan et al.  2020[17] | Yoon et al  2018[18] | Wu et al.  2025[19] | Liu et al.  2018[20] | Ye et al.  2021[21] | Chen et al.  2021[22] | Falck et al.  2025[23] | Zhu et al.2023[24] |
| Screening questions  (for all types) | S1. Are there clear research questions? | √ | √ | √ | √ | √ | √ | √ | √ |
|  | S2. Do the collected data allow to address the research questions? | √ | √ | √ | √ | √ | √ | √ | √ |
|  | *Further* *appraisal* *may* *not* *be feasible* *or* *appropriate* *when* *the* *answer* *is* *‘No’* *or* *‘Can’t* *tell’* *to* *one* *or* *both* *screening* *questions.* | | | | | | | | |
| **2.Quantitative randomized controlled trials** | 2.1. Is randomization appropriately performed? | √ | √ | √ | √ | √ | √ | √ | √ |
|  | 2.2. Are the groups comparable at baseline? | √ | √ | √ |  | √ | √ | √ | √ |
|  | 2.3. Are there complete outcome data? | √ | √ | √ | √ | √ | √ | √ | √ |
|  | 2.4. Are outcome assessors blinded to the intervention provided? |  |  | √ |  | √ | √ | √ |  |
|  | 2.5 Did the participants adhere to the assigned intervention? | √ | √ | √ | √ | √ | √ | √ | √ |
|  | Quality evaluation results | **** | **** | ***** | *** | ***** | ***** | ***** | **** |

| **Category of study designs** | **Methodological quality criteria** | Study | | | | | | | |
| --- | --- | --- | --- | --- | --- | --- | --- | --- | --- |
|  |  | Jia et al  2022[25] | Wan et al.  2022[26] | Lin et al.  2023[27] | Ye et al.  2024[28] | Wang et al.  2024[29] | Yang et al.  2023[30] | Kwan et al.  2021[31] | Lai et al.  2025[32] |
| Screening questions  (for all types) | S1. Are there clear research questions? | √ | √ | √ | √ | √ | √ | √ | √ |
|  | S2. Do the collected data allow to address the research questions? | √ | √ | √ | √ | √ | √ | √ | √ |
|  | *Further* *appraisal* *may* *not* *be feasible* *or* *appropriate* *when* *the* *answer* *is* *‘No’* *or* *‘Can’t* *tell’* *to* *one* *or* *both* *screening* *questions.* | | | | | | | | |
| **2.Quantitative randomized controlled trials** | 2.1. Is randomization appropriately performed? | √ | √ | √ | √ | √ | √ | √ | √ |
|  | 2.2. Are the groups comparable at baseline? | √ | √ | √ | √ | √ | √ | √ | √ |
|  | 2.3. Are there complete outcome data? | √ | √ | √ | √ | √ | √ | √ | √ |
|  | 2.4. Are outcome assessors blinded to the intervention provided? |  | √ | √ |  | √ |  | √ |  |
|  | 2.5 Did the participants adhere to the assigned intervention? | √ | √ | √ | √ | √ | √ | √ | √ |
|  | Quality evaluation results | **** | ***** | ***** | **** | ***** | **** | ***** | **** |

continued

| **Category of study designs** | **Methodological quality criteria** | Study |
| --- | --- | --- |
|  |  | Liao et al.2025[33] |
| Screening questions  (for all types) | S1. Are there clear research questions? | √ |
|  | S2. Do the collected data allow to address the research questions? | √ |
|  | *Further* *appraisal* *may* *not* *be feasible* *or* *appropriate* *when* *the* *answer* *is* *‘No’* *or* *‘Can’t* *tell’* *to* *one* *or* *both* *screening* *questions.* | |
| **3. Quantitative nonrandomized** | 3.1. Are the participants representative of the target population? | √ |
|  | 3.2. Are measurements appropriate regarding both the outcome and intervention (or exposure)? | √ |
|  | 3.3. Are there complete outcome data? | √ |
|  | 3.4. Are the confounders accounted for in the design and analysis? |  |
|  | 3.5. During the study period, is the intervention administered (or exposure occurred) as intended? | √ |
|  | Quality evaluation results | **** |
